# Correcting AUC for Measurement Error

**DOI:** 10.4172/2155-6180.1000270

**Published:** 2015-12-28

**Authors:** Bernard Rosner, Shelley Tworoger, Weiliang Qiu

**Affiliations:** 1 Channing Division of Network Medicine, Brigham and Women's Hospital, Harvard Medical School, 181 Longwood Avenue, Boston, MA 02115, USA; 2 Department of Biostatistics, Harvard T.H. Chan School of Public Health, 655 Huntington Avenue, Boston, Massachusetts 02115, USA; 3 Department of Epidemiology, Harvard T.H. Chan School of Public Health, 677 Huntington Avenue Boston, Massachusetts 02115, USA

**Keywords:** AUC, Biomarkers, Non-normal distributions

## Abstract

Diagnostic biomarkers are used frequently in epidemiologic and clinical work. The ability of a diagnostic biomarker to discriminate between subjects who develop disease (cases) and subjects who do not (controls) is often measured by the area under the receiver operating characteristic curve (AUC). The diagnostic biomarkers are usually measured with error. Ignoring measurement error can cause biased estimation of AUC, which results in misleading interpretation of the efficacy of a diagnostic biomarker. Several methods have been proposed to correct AUC for measurement error, most of which required the normality assumption for the distributions of diagnostic biomarkers. In this article, we propose a new method to correct AUC for measurement error and derive approximate confidence limits for the corrected AUC. The proposed method does not require the normality assumption. Both real data analyses and simulation studies show good performance of the proposed measurement error correction method.

## Introduction

Diagnostic biomarkers are used frequently in epidemiologic and clinical work. The ability of a diagnostic biomarker to discriminate between subjects who develop disease (cases) and subjects who do not (controls) is often measured by the area under the receiver operating characteristic curve (AUC), with values close to 1.0 indicating high diagnostic accuracy. The AUC can be interpreted as

$$AUC_{\text{obs}}=Pr(Y_{\text{obs}}<X_{\text{obs}})$$

where X_obs_ is the value of the diagnostic biomarker for a randomly selected case and Y_obs_ is the value of the diagnostic biomarker for a randomly selected control. AUC takes values between 0.5 and 1. AUC close to 0.5 indicates no diagnostic accuracy; AUC close to 1.0 indicates high diagnostic accuracy.

Under the normality assumption that $X_{i}∼N(\mu_{X,\text{obs}},\sigma_{X,\text{obs}}^{2}),Y_{j}∼N(\mu_{Y,\text{obs}},\sigma_{Y,\text{obs}}^{2}),$ and X_i_ and Y_j_, i = 1,…,m, j = 1,…,n, are all independent, AUC is calculated as [1]:

$$AUC_{\text{obs}}=\Phi (\delta_{\text{obs}})$$

where

$$\delta_{\text{obs}}=\frac{\mu_{X,\text{obs}}-\mu_{Y,\text{obs}}}{\sqrt{\sigma_{X,\text{obs}}^{2}+\sigma_{Y,\text{obs}}^{2}}}$$

It is extensively documented in the medical literature that diagnostic biomarkers may be subject to errors of measurement [2], which may be attributed to variation in performance of laboratory equipment, variation between technicians, temporal changes, biologic variability, etc. It has been reported [1,2] that ignoring measurement error can cause biased estimation of AUC. In many cases, the biases can result in misleading interpretation of the efficacy of a diagnostic biomarker [3]. For example, not adjusting for measurement error can result in useful diagnostic biomarkers being overlooked. In general, an increase in measurement error moves the receiver operating characteristic (ROC) curve towards the diagonal (non-informative) line, and the value of the AUC is decreased [4,5].

The biases of estimators usually can be corrected by resampling methods (e.g., jackknife or bootstrap). However, resampling methods are not appropriate when biases are caused by non-sampling errors, such as measurement error [2]. Several methods [1- 3,6] have been proposed in the literature to correct estimates of the AUC when accounting for measurement error. Coffin and Sukhatme [1] and Coffin and Sukhatme [2] assumed the following measurement error model:

(1) $$\begin{array}{cc} X_{i,\text{obs}}=X_{i,\text{true}}+{ɛ}_{i}, & i=1,\ldots ,m,\\ Y_{j,\text{obs}}=Y_{j,\text{true}}+\xi_{j}, & j=1,\ldots ,n, \end{array}$$

where

$$\begin{array}{l} X_{i,\text{true}}\sim F_{X,\text{true}}(\mu_{X},\sigma_{X}^{2}),{ɛ}_{i}\sim F_{ɛ}(0,\sigma_{ɛ}^{2}),\\ Y_{j,\text{true}}\sim F_{Y,\text{true}}(\mu_{Y},\sigma_{Y}^{2}),\xi_{j}\sim F_{\xi }(0,\sigma_{\xi }^{2}), \end{array}$$

F (a,b) is a cumulative distribution function (CDF) with mean *a* and variance b, and X_i,true_, Y_j,true_, *ε_i_*, and *ξ_j_*, i = 1,…,m, j = 1,…,n, are mutually independent. F_Y,true_, AUC_obs_

Coffin and Sukhatme (1995) [1] assumed F_x,true_, F_Y,true_, *F_ε_*, and *F_ξ_* are CDFs from an exponential family and derived an approximate bias *C* of the observed AUC due to measurement error and then obtained estimates of the corrected AUC by adding this bias term to the observed AUC, i.e., *AUC_corrected_ ≈ AUC_obs_ +C*. Coffin and Sukhatme's [1] Monte Carlo simulation studies showed that the bias of the corrected AUC (AUC_corrected_) is generally an order of magnitude smaller than the bias of the AUC without measurement error correction (AUC_obs_). Also the corrected AUC estimate (AUC_corrected_) has comparable mean square error (MSE) to AUC_obs_. Coffin and Sukhatme [2] noted that the AUC estimated by the Mann-Whitney U statistic is also subject to measurement error. Paralleling to Coffin and Sukhatme [1], Coffin and Sukhatme [2] used a non-parametric approach to derive an approximate bias C for the AUC estimated by the Mann-Whitney U statistic. The simulation studies in Coffin and Sukhatme [2] showed that for several families of distributions (normal, gamma, or t distributions), bias-corrected AUC have much smaller bias and comparable MSE to the AUC estimated by the Mann-Whitney U statistic.

Faraggi [3] derived an exact relationship between the observed AUC and the true AUC by assuming that F_x,true_, F_Y,true_, *F_ε_*, and *F_ξ_* are CDFs of normal distributions and by assuming equal variance (i.e., $\sigma_{e}^{2}=\sigma_{ɛ}^{2}=\sigma_{\xi }^{2}$ and $\sigma^{2}=\sigma_{X}^{2}=\sigma_{Y}^{2}$), whereby

(2) $$AUC_{\text{true}}=\Phi \{\Phi^{-1}(AUC_{\text{obs}})\sqrt{1+\theta^{2}}\},$$

where $\theta^{2}=\sigma_{e}^{2}/\sigma^{2}$. Faraggi [3] also derived a 95% confidence interval (CI) for AUC_true_ when *θ*^2^ is known. Faraggi [3] showed numerically that not taking measurement error into account can give seriously misleading results that understate the diagnostic effectiveness (i.e., the coverage probability of the unadjusted confidence interval can be far from its nominal value when measurement error is present).

The method proposed by Faraggi [3] requires that the ratio *θ*^2^ of intra-individual to inter-individual variation was accurately known (e.g., based on prior experience). If *θ*^2^ is unknown, either repeated measurement or an external validation study is required to estimate *θ*^2^. Reiser [6] generalized the formula for *θ*^2^ by allowing different variances and provided an estimate of *θ*^2^ based on repeated measurements X_ik,obs_ and Y_j_*_l_*_,obs_, where the subscripts k and *l* indicate the *k*-th and *l*-th replicates for the *i*-th case and the *j*-th control, respectively. The measurement error model that Reiser [6] assumed is

(3) $$\begin{array}{c} X_{ik,\text{obs}}=X_{i,\text{true}}+{ɛ}_{ik},\\ Y_{j\ell ,\text{obs}}=Y_{j,\text{true}}+\xi_{j\ell }, \end{array}$$

where

$$\begin{array}{l} X_{i,\text{true}}\sim N(\mu_{X},\sigma_{X}^{2}),{ɛ}_{ik}\sim N(0,\sigma_{ɛ}^{2}),\\ Y_{j,\text{true}}\sim N(\mu_{Y},\sigma_{Y}^{2}),\xi_{j\ell }\sim N(0,\sigma_{\xi }^{2}), \end{array}$$

and *X_i,true_*, *Y_j,true_*, *ε_ik_* and *ξ_jℓ_*, *i* = 1,…,*m*, *k* = 1,…,*m_i_*, *j* = 1,…,*n, ℓ* = 1,…,*n_j_* are mutually independent. Based on (??), it follows that

(4) $$AUC_{\text{true}}=\Phi (\delta_{\text{true}}),\delta_{\text{true}}=(\mu_{X}-\mu_{Y})/\sqrt{\sigma_{X}^{2}+\sigma_{Y}^{2}}.$$

The relationship between AUC_true_ and AUC_obs_ again has the form (??), where

$$\theta^{2}=(\sigma_{ɛ}^{2}+\sigma_{\xi }^{2})/(\sigma_{X,\text{true}}^{2}+\sigma_{Y,\text{true}}^{2}).$$

Reiser [6] used the delta method to obtain the approximate variance of the estimate *δ̂_true_*, then obtained the 95% CI for *δ_true_* and *AUC_true_* = Φ (*δ_true_*).

Li et al. [7] provided an alternative method to obtain the variance of the estimate *δ̂_true_* by using the method of variance estimates recovery (MOVER), which allows the variance estimate to change with the underlying parameter values.

Schisterman et al. [4] proposed a AUC correction method for the case where no repeated measurements are available, but an external validation data set is available. In addition to the normality assumption, Schisterman et al. [4] assumed that the distributions in the external validation data set are the same as those in the main study. Li et al.[7] method can also be used for the case where an external validation data set is available.

Tosteson et al. [5] extended the measurement error model (??) by assuming that F_x,true_, and F_Y,true_, are CDFs of normal distributions, but the error terms *ε_i_* and *ξ_j_* have non-normal distributions. They derived the measurement error correction for sensitivity, specificity, and sensitivity at a given value of specificity, but not for AUC.

Most of the aforementioned AUC measurement correction methods require the normality assumption. However, the normality assumption is often violated in real data analysis. Some of these methods assumed the location-shift hypothesis:

$$F_{X,\text{true}}(z)=F_{Y,\text{true}}(z-\eta ),$$

for *η* ≠ 0, where F_x,true_, and F_Y,true_, are the cumulative distribution functions of the biomarker for cases and controls, respectively. The location-shift hypothesis is reasonable for symmetric distributions, but may not be ideal for skewed distributions as the mean is no longer a good summary of the distribution center.

In this paper, we aim to extend the method of Reiser [6] by relaxing the normality assumption. The paper is arranged as follows: In section 2, we first present a measurement-error-correction method for AUC under the probit-shift hypothesis without requiring the normality assumption. We then construct confidence intervals for the corrected AUC. In Section 3, we present a simulation study. In Section 4, we present results from data analysis of a real example based on the Swiss Analgesic Study. Section 5 is a discussion.

## Methods

### AUC for non-normally distributed diagnostic biomarkers measured without error

We first consider how to handle the non-normality for a diagnostic biomarker *M* measured without error. We propose a probit-shift model

(5) $$\Phi^{-1}\{F_{Y,\text{true}}(z)\}=\Phi^{-1}\{F_{X,\text{true}}(z)\}+\mu ,$$

or equivalently

$$F_{Y,\text{true}}(z)=\Phi [\Phi^{-1}\{F_{X,\text{true}}(z)\}+\mu ],$$

where Φ is the CDF of the standard normal distribution. That is, after probit transformations, the distributions of cases and controls satisfy the location-shift property.

Thus, the AUC is a function of *μ*. If we let *w* = *H_X_*(*x*) *≡* Φ^−1^{*F_X_*(*x*)} then based on (??) it follows that

$$\begin{array}{ll} AUC_{\text{true}}(\mu )= & Pr(Y_{\text{true}}<X_{\text{true}})\\ = & \int_{x=-\infty }^{\infty }F_{Y,\text{true}}(x)f_{X,\text{true}}(x)dx\\ = & \int_{w=-\infty }^{\infty }\Phi (w+\mu )\phi (w)dw. \end{array}$$

We can use a first order Taylor series approximation to approximate the above integration (c.f Online Supplementary Document Section A, Equation A1) and obtain:

$$AUC_{\text{true}}(\mu )\approx \Phi (\frac{\mu }{\sqrt{2}}).$$

### AUC for non-normally distributed diagnostic biomarkers measured with error

We assume the following measurement error model for probit transformed data:

(6) $$\begin{array}{l} H_{X,\text{obs}}(z)=H_{X,\text{true}}(z)+e_{X},e_{X}\sim N(0,\sigma_{e_{X}}^{2}),\\ H_{Y,\text{obs}}(z)=H_{Y,\text{true}}(z)+e_{Y},e_{Y}\sim N(0,\sigma_{e_{Y}}^{2}),\\ H_{Y,\text{true}}(z)=H_{X,\text{true}}(z)+\mu \end{array}$$

where *H_X,true_* (*z*) = Φ^−1^ {*F_X,true_* (*z*)}, *H_X,obs_* (*z*) = Φ^−1^ {*F_X,obs_* (*z*)}, *e_X_* is independent of *H_X,true_*, and *H_Y,true_* and *H_Y,obs_* are defined similarly. *F_X,true_* (*z*), *F_X,obs_* (*z*), *F_Y,true_* (*z*), and *F_Y,obs_* (*z*) are the cumulative distribution functions of the underlying true/observed values of the diagnostic biomarker *M*, respectively. We assume that *e_X_* and *e_Y_* are independent.

To derive the relationship between the true AUC and the observed AUC, we first consider the conditional observed AUC:

$$\begin{array}{ll} AUC_{\text{obs}}(\mu )|e_{Y},e_{X}= & Pr(Y_{\text{obs}}<X_{\text{obs}}|e_{Y},e_{X})\\ = & \int_{x=-\infty }^{\infty }F_{Y,\text{obs}}(x)dF_{X,\text{obs}}(x). \end{array}$$

Note that

$$\Phi^{-1}\{F_{Y,\text{obs}}(x)\}=H_{X,\text{true}}(x)+\mu +e_{Y}.$$

Hence,

$$AUC_{\text{obs}}(\mu )|e_{Y},e_{X}=\int_{x=-\infty }^{\infty }\Phi \{H_{X,\text{true}}(x)+\mu +e_{Y}\}dF_{X,\text{obs}}(x).$$

Note that

$$\begin{array}{l} F_{X,\text{obs}}(x)=\Phi \{H_{X,\text{true}}(x)+e_{X}\},\\ dF_{X,\text{obs}}(x)=\phi \{H_{X,\text{true}}(x)+e_{X}\}dH_{X,\text{true}}(x). \end{array}$$

Thus,

$$AUC_{\text{obs}}(\mu )|e_{Y},e_{X}=\int_{x=-\infty }^{\infty }\Phi \{H_{X,\text{true}}(x)+\mu +e_{Y}\}\phi \{H_{X,\text{true}}(x)+e_{X}\}dH_{X,\text{true}}(x)$$

Upon integration and use of the delta method (c.f. Online Supplementary Document Equation (A7))

$$AUC_{\text{obs}}(\mu )\approx \Phi [\frac{\Phi^{-1}\{AUC_{\text{true}}(\mu )\}}{\sqrt{\left(1+\frac{\sigma_{e_{X}}^{2}+\sigma_{e_{Y}}^{2}}{2}\right)}}]$$

or equivalently, based on Online Supplementary Document Equation (A9),

(7) $$AUC_{\text{true}}(\mu )\approx \Phi [\Phi^{-1}\{AUC_{\text{obs}}(\mu )\}\times \sqrt{\frac{\frac{1}{ICC_{X}}+\frac{1}{ICC_{Y}}}{2}}].$$

where *ICC_X_* and *ICC_Y_* are intra-class correlations

$$ICC_{X}=\frac{1}{1+\sigma_{e_{X}}^{2}},ICC_{Y}=\frac{1}{1+\sigma_{e_{Y}}^{2}}.$$

We assume there exists at least one replicated observation for each subject in the data set or in a subset of the data set and that the replicates are distinguishable, so that we can determine unique probit scales for each subject and each replicate and then can estimate the intra-class correlations *ICC_X_* and *ICC_Y_* by using the variance components from a one-way ANOVA. We used the function ICCest of the package ICC[8] from the statistical software R[9] to calculate ICCs. Furthermore, because the probit transformation is a rank-invariant transformation, we can use the Mann-Whitney statistic to estimate *AUC_obs_*(*μ*) [10] (c.f Formula A13 in the Online Supplementary Document Section D.1). When we estimate *AUC_obs_* (*μ*), only the data in the main study were used (replicates were not used). Replicates were used only to estimate ICCs.

The relationship (??) between *AUC_true_* (*μ*) and *AUC_obs_* (*μ*) provides a method to correct measurement error for the observed *AUC_obs_*(*μ*). Hence, we also refer to *AUC_true_* (*μ*) as the corrected AUC and denote it as AUC_corrected_.

## Confidence limits for *AUC_true_* (*μ*)

We use the delta method to derive the variance of the true AUC. Denote

$$a=\Phi^{-1}(AUC_{\text{obs}}(\mu )),b=\sqrt{\frac{\frac{1}{ICC_{x}}+\frac{1}{ICC_{y}}}{2}.}$$

We have

$$AUC_{\text{true}}(\mu )=\Phi (a\times b)$$

An approximate 100%×(1−*α*) CI for *AUC_true_* (*μ*) is given by {Φ(*c*_1_), Φ(*c*_2_)}, where

(8) $$(c_{1},c_{2})=[(\hat{a}\times \hat{b})-z_{1-\alpha /2}se(\hat{a}\times \hat{b}),(\hat{a}\times \hat{b})+z_{1-\alpha /2}se(\hat{a}\times \hat{b})],$$

The detailed derivations of *c*_1_ and *c*_2_ are shown in the online supplementary document Sections C and D.

## A Simulation Study

To evaluate the performance of the proposed AUC estimate *ÂUC_true_* (*μ̂*) that corrects for measurement error, we conducted 3 simulation studies. In each simulation study, we generated 1000 simulated data sets, each of which contains 100 cases and 100 controls. We then ran each simulation study 100 times to obtain the mean performance measure over the 100 simulations and to estimate the 95% confidence interval (CI) of the performance measures, such as bias, mean square error (MSE), and coverage.

We also compared the performance of AUC_corrected_ in (??) with that proposed by Reiser [6] in equation (??). Both methods require the availability of replicate observations.

### Simulation model I

In the first simulation study, we assumed that there are replicate observations for each subject and generated simulated data using Reiser's [6] model (c.f. Formula (??)). That is, *X_i,true_, Y_j,true_, e_Xi_* and *e_Yj_* were generated from normal distributions. To generate replicates, we generated another set of error terms *e_X_i′__* and *e_Y_j′__*, but kept the values of true observations *X_i,true_*, *Y_j,true_*, so that the 2 observations for the same subject would be dependent.

### Simulation model II

In the second simulation study, we assumed that *X_i,true_* and *Y_j,true_* were from log-normal distributions, while the error terms *ε_X,i_* and *ε_Y,j_* were from normal distributions:

(9) $$\begin{array}{l} X_{i,\text{obs}}=X_{i,\text{true}}+{ɛ}_{X,i},\log(X_{i,\text{true}})∼N(\lambda +\mu ,\sigma_{X,\text{true}}^{2}),{ɛ}_{X,i}∼N(0,\sigma_{{ɛ}_{X}}^{2}),\\ Y_{j,\text{obs}}=Y_{j,\text{true}}+{ɛ}_{Y,i},\log(Y_{j,\text{true}})∼N(\lambda ,\sigma_{Y,\text{true}}^{2}),{ɛ}_{Y,i}∼N(0,\sigma_{{ɛ}_{Y}}^{2}),\\ i=1,\ldots ,m,j=1,\ldots ,n. \end{array}$$

To generate replicates, we generated another set of error terms *ε_X_i′__* and *ε_Y_j′__*, but kept the values of true observations *X_i,true_*, *Y_j,true_*, so that the 2 observations for the same subject would be dependent.

### Simulation model III

In the third simulation study, we assumed that *X_i,true_* and *Y_j,true_ ε_X,i_* and *ε_Y,j_* were all from log-normal distributions:

(10) $$\begin{array}{l} X_{i,\text{obs}}=X_{i,\text{true}}+{ɛ}_{X,i},\log(X_{i,\text{true}})∼N(\lambda +\mu ,\sigma_{X,\text{true}}^{2}),\log({ɛ}_{X,i})∼N(0,\sigma_{{ɛ}_{X}}^{2}),\\ Y_{j,\text{obs}}=Y_{j,\text{true}}+{ɛ}_{Y,i},\log(Y_{j,\text{true}})∼N(\lambda +\sigma_{Y,\text{true}}^{2}),\log({ɛ}_{Y,i})∼N(0,\sigma_{{ɛ}_{Y}}^{2}),\\ i=1,\ldots ,m,j=1,\ldots ,N. \end{array}$$

To generate replicates, we generated another set of error terms *ε_X,i′_* and *ε_Y,j′_*, but kept the values of true observations *X_i,true_, y_j,true_,* so that the 2 observations for the same subject would be dependent.

### Parameter settings

For Simulation Model I, the true AUC value is *AUC_true_* = Φ(*δ*), where $\delta =(\mu_{X}-\mu_{Y})/(\sqrt{\sigma_{X,\text{true}}^{2}+\sigma_{Y,\text{true}}^{2}})$. We set *m* = *n* = 100, *m_i_* = *n_j_* = 2, $\sigma_{X,\text{true}}^{2}=\sigma_{Y,\text{true}}^{2}=1,\sigma_{ɛ}^{2}=\sigma_{\eta }^{2}=0.5$, *μ_Y_* = 0, and *μ_X_* = 0.25,0.5, or 1.

For Simulation Models II and III, we can show that (c.f. Online Supplementary Document Section E) $AUC_{\text{true}}=Pr(Y_{\text{true}}<X_{\text{true}})=\Phi \{\mu_{X}/\sqrt{\sigma_{X,\text{true}}^{2}+\sigma_{Y,\text{true}}^{2}}\}$. We set *m* = *n* = 100, *λ* = 0, $\sigma_{X,\text{true}}^{2}=\sigma_{Y,\text{true}}^{2}=\sigma_{{ɛ}_{X}}^{2}=\sigma_{{ɛ}_{X}}^{2}=1$, *μ_Y_* = 0, and *μ_X_* = 0.25, 0.5, or 1.

For Simulation Models I, II, and II, the true AUC values are 0.57 (for *μ* = *μ_X_* − *μ_Y_* = 0.25), 0.64 (for *μ* = *μ_X_* − *μ_Y_* = 0.5), and 0.76 (for *μ* = *μ_X_* − *μ_Y_* = 1), respectively.

To evaluate the effects of sample size and unequal variance on the performances of the three methods, we also performed an addtional set of simulations with *m* = *n* = 50 and $\sigma_{X,\text{true}}^{2}/\sigma_{Y,\text{true}}^{2}=2(\sigma_{X,\text{true}}^{2}=2,\sigma_{X,\text{true}}^{2}=1)$ and the same set of other parameters as above.

To further evaluate the effect of the value of $\theta^{2}=(\sigma_{ɛ}^{2}+\sigma_{\xi }^{2})/(\sigma_{X,\text{true}}^{2}+\sigma_{Y,\text{true}}^{2})$ (i.e., the degree of measurement error), we performed another set of simulations with *m* = *n* = 50, $\sigma_{X,\text{true}}^{2}+\sigma_{Y,\text{true}}^{2}=2(\sigma_{X,\text{true}}^{2}=2,\sigma_{Y,\text{true}}^{2}=1)$, and $\theta^{2}=3(\sigma_{ɛ}^{2}=\sigma_{\xi }^{2}=4.5)$.

### Results of simulation studies

Tables 1-3 and online supplementary Figure 1 summarized the results of the three simulation studies. We observed that (1) the observed (i.e., uncorrected) AUC estimates *AUC_obs_* underestimated the true AUC for all 9 scenarios (i.e., the estimated biases were negative and the estimated coverages were less than the nominal value 0.95); (2) The MSE of *AUC_obs_* was much larger than those of the proposed method and Reiser's method when *μ* = 1; (3) as the value of *μ* increases, the absolute bias and MSE generally increased for all 3 types of AUC estimates; (4) for Simulation Study I (i.e., data were generated under Reiser's model), the probit method had similar performance to Reiser's method; (5) for Simulation Studies II and III (i.e., data were generated from non-normal distributions), the coverages estimated by the proposed method were close to the nominal value 0.95, while the coverages of the uncorrected AUC and the coverages of the corrected AUC estimated by Reiser's method were smaller than the nominal value, especially when the value of *μ* was large; (6) for Simulation Studies II and III, the proposed method had much smaller absolute bias than the other two methods.

Tables S1, S2, and S3 in the online Supplementary Documents showed the results for the simulations with smaller sample size *m* = *n* = 50 and with unequal variance $\sigma_{X,\text{true}}^{2}=2$ and $\sigma_{Y,\text{true}}^{2}=1$. The results are similar to those shown in Tables 1-3.

If the degree of measurement error as characterized by $\theta^{2}=(\sigma_{ɛ}^{2}+\sigma_{\xi }^{2})/(\sigma_{X,\text{true}}^{2}+\sigma_{Y,\text{true}}^{2})$ is large (*θ*^2^ = 3 say), the bias of the probit method is smaller than the other two approches. However, the coverage of Reiser's method and the probit-shit method tend to be somewhat larger than the nominal level 0.95 (c.f, Tables S4, S5, S6 in the online Supplementary Documents).

## Examples

In this section, we used a real data set (the Swiss Analgesic Study data set) to evaluate the performance of the proposed measurement correction method for AUC estimation.

The Swiss Analgesic Study data set was collected starting from 1967/1968 [11]. There were 1244 Swiss women participating in this study whose purpose was to evaluate the association of the use of phenacetin-containing analgesics with kidney function. NAPAP is a biomarker which is associated with recent use of phenacetin-containing analgesics. The NAPAP value was measured in a urine sample at the baseline clinic visit. There were additional follow-up collections of NAPAP values at home on 2 separate days within 1 week of the baseline clinic visit. In addition, serum creatinine was measured at the baseline clinic visit.

**Figure 1:**
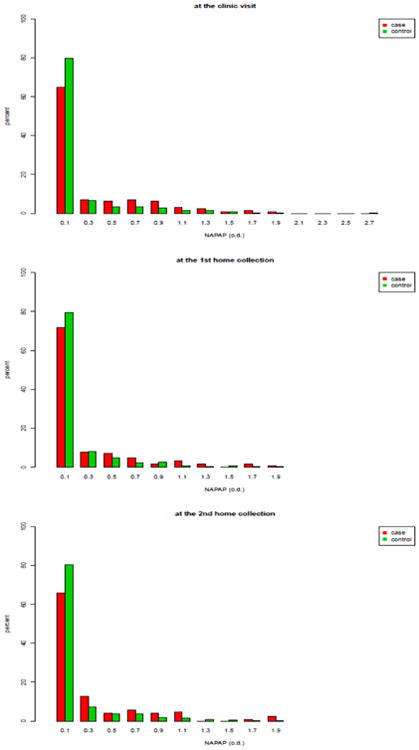
Histograms of the NAPAP values. The upper panel: cases (left) and controls (right) measured at the clinic visit; The middle panel: cases (left) and controls (right) measured at the first home collection; The bottom panel: cases (left) and controls (right) measured at the second home collection.

We wish to investigate whether excessive recent intake of phenacetin-containing analgesics as determined by the urinary NAPAP level can be used as a screening test for identifying subjects with abnormal kidney function as determined by elevated serum creatinine. For this purpose, we dichotomized the baseline serum creatinine level. If a woman had elevated baseline serum creatinine (i.e., serum creatinine ≥1.5*mg* / *dL*), she was classified as a case; otherwise she was classified as a control. There were 1081 controls, 128 cases, and 35 subjects missing baseline serum creatinine. In the analysis, 1209 women without missing values were used. We would like to assess if NAPAP values could be used to discriminate between cases and controls. The AUC based on the NAPAP values measured at the clinic visit was used to measure the discrimination ability of the NAPAP assay. The 3 replicates were used to calculate ICC values. By examining the histograms of the NAPAP values for cases and controls, we found the distribution of the NAPAP value is quite skewed in both cases and controls in all 3 measurements (Figure 1). Hence, the normality assumption was violated.

The estimated AUC and 95% confidence interval (CI) of AUC are summarized in Table 4. The estimated AUC based on the Mann-Whitney U statistic (i.e., the uncorrected estimate of AUC) was 0.589 with 95% confidence interval (CI) [0.537,0.640]. The corrected AUC estimate based on Reiser's [6] method was 0.611 with 95% CI [0.557,0.663]. The corrected AUC estimate based on the probit-shift method was 0.618 with 95% CI [0.549,0.684]. In this example, the number of women with replicated observations is 1193, the estimated ICC based on probit transformed data was 0.648 for cases and 0.498 for controls. Hence, the corrected AUC is similar for the Reiser's and probit-shift methods, but the confidence limits are wider for the latter method.

## Discussion

In this article, we presented a method to correct AUC for measurement error without making the assumption of normally distributed diagnostic biomarkers. Instead, we use the probit transformation to create a transformed diagnostic biomarker, which on the probit scale is approximately normally distributed. To implement our approach, one needs replicate data on at least a subsample of subjects to compute the intraclass correlation. The replicates should be close enough in time so that the assumption that the underlying mean diagnostic biomarker level is the same is not violated. Simulation studies support the validity of the methods based on moderate sized samples of 100 cases and 100 controls.

The simulation studies demonstrated that without correcting for measurement error would result in AUC biased toward the null value (0.5) Under the normality assumption, the proposed method has similar performance as Reiser's method which requires the normality assumption in measurement error modelling. When the normality assumption is violated, the proposed method performed much better than Reiser's method in terms of bias and coverage.

The probit-shift model assumes equal variance $\sigma_{X,\text{true}}^{2}=\sigma_{Y,\text{true}}^{2}$. In the simulation studies, we evaluated the effects of unequal variance on the performance of the probit-shift model. The results were similar to Tables 1-3, if measurement error is small as characterized by *θ*^2^. If *θ*^2^ > 1, then the probit-shift model still has minimal bias, but has observed coverage greater than nominal coverage. In future work, we will extend the probit-shift model to allow unequal variance scenario, in which the probit-shift model would have the following form:

$$H_{Y,\text{true}}(z)=c_{1}H_{X,\text{true}}(z)+c_{2},$$

where *c*_1_ = *σ_x,true_* / *σ_y,true_* and *c*_2_ = (*μ_X,true_* − *μ_Y,true_*) / *σ_Y,true_*.

In the real data analysis, the corrected AUC by the proposed method was similar to the corrected AUC by the Reiser's method, although the distributions of the biomarker in both cases and controls were highly skewed. This is probably because the unknown true AUC is close to the null value 0.5. The three simulation studies also demonstrated this point. That is, when *μ* is close to 0 or equivalently when *AUC_true_* is close to 0.5, the 3 AUC estimation methods gave similar results. However, confidence limits are wider with the probit-shift method.

An implicit assumption of our approach is that the distribution of diagnostic biomarkers is continuous. If instead, risk is defined based on a limited number of categorical risk factors, then the diagnostic biomarker distribution will be discrete and the assumption that the probit transformation results in a normally distributed scale will only be approximately satisfied and needs to be studied in more detail.

It is worth noting that several authors have developed measurement-error-correction approaches for estimating a variety of diagnostic performance measures other than AUC, including sensitivity, specificity, and the Youden index [12]. The probit-shift method may be useful in incorporating the effects of measurement error on these indices in the setting of non-normally distributed diagnostic biomarkers.

## Supplementary Material

Supplementary file

## Figures and Tables

**Table 1:** Bias, mean square error (MSE), and coverage for *AUC_true_* (*μ*) from simulation I **.

λ	μ_Y_	μ_X_	AUC_true_		MW*	R*	P*
0	0	0.25	0.570	Bias(×10^3^) 95% Cl	-13 (-17, -9)	0 (-4,4)	0 (-4,5)
				MSE(×10^4^) 95% Cl	18 (16,20)	19 (18,22)	25 (22,28)
				Coverage (%) 95% Cl	93.7 (91.8,95.6)	95.1 (93.4, 96.9)	94.9 (93.0, 96.8)
0	0	0.5	0.638	Bias(×10^3^) 95% Cl	-25 (-28, -21)	0 (-4,4)	0 (-4,5)
				MSE(×10^4^) 95% Cl	22 (19,24)	18 (16,20)	23 (20,26)
				Coverage(%) 95% Cl	90.3 (87.6, 93.0)	95.2 (93.5, 96.8)	94.8 (92.9, 96.7)
0	0	1	0.760	Bias(×10^3^) 95% Cl	-42 (-45, -39)	0 (-4,3)	0 (-3,4)
				MSE(×10^4^) 95% Cl	31 (27,34)	14 (12,16)	17 (15,20)
				Coverage (%) 95% Cl	76.6 (73.0, 80.2)	95.2 (93.3, 97.0)	94.4 (92.4, 96.5)

* MW: Mann-Whitney estimate (i.e., *AUC_obs_*); R: Reiser's (2000) method; P: Probit method.

** Simulation I was run 100 times. Each time, we generated 1000 simulated data sets. Each data set consists of 100 cases and 100 controls. Each subject provides two replicate biomaker scores. Both true values and random errors are assumed to come from normal distributions with $\sigma_{X,\text{true}}^{2}=\sigma_{Y,\text{true}}^{2}=1,\sigma_{ɛ}^{2}=\sigma_{\eta }^{2}=0.5$.

**Table 2:** Bias, mean square error (MSE), and coverage for *AUC_true_* (*μ*) from simulation II**.

λ	μ_Y_	μ_X_	AUC_true_		MW*	R*	P*
0	0	0.25	0.57	Bias(×10^3^) 95% Cl	-23 (-26, -19)	15 (-18, -11)	4 (-9,1)
				MSE(×10^4^) 95% Cl	22 (19, 25)	20 (17,22)	32 (28,26)
				Coverage (%) 95% Cl	91.2 (88.5,93.9)	93.8 (91.6, 95.6)	94.8 (92.8, 96.8)
0	0	0.50	0.638	Bias(×10^3^) 95% Cl	- 49 (-52, -45)	-32 (-35, -28)	-7 (-12, -2)
				MSE(×10^4^) 95% Cl	39 (35,44)	26 (23,29)	36 (31,41)
				Coverage (%) 95% Cl	76.3 (72.1,80.5)	89.6 (86.9, 92.2)	94.7 (92.7, 96.8)
0	0	1.0	0.760	Bias(×10^3^) 95% Cl	-104 (-107, -101)	-74 (-77, -70)	2 (-4, 8)
				MSE(×10^4^) 95% Cl	122 (115, 130)	69 (64,74)	53 (46, 61)
				Coverage (%) 95% Cl	17.4 (14.6, 20.3)	52.3 (47.6, 56.9)	95.3 (93.1, 97.5)

* MW: Mann-Whitney estimate (i.e., *AUC_obs_*); R: Reiser's (2000) method; P: Probit method.

** Simulation II was run 100 times. Each time, we generated 1000 simulated data sets. Each data set consists of 100 cases and 100 controls. Each subject provides two replicate biomaker scores. True values were generated from log normal distributions and random errors were generated from normal distributions with $\sigma_{X,\text{true}}^{2}=\sigma_{Y,\text{true}}^{2}=\sigma_{e_{X}}^{2}=\sigma_{e_{Y}}^{2}=1$.

**Table 3:** Bias, mean square error (MSE), and coverage for *AUC_true_* (*μ*) from simulation III**.

A	μ_Y_	μ_X_	AUC^true^		MW*	R*	P*
0	0	0.25	0.570	Bias(×10^3^) 95% Cl	- 26 (-29, -22)	- 14 (-19, -10)	0 (-6,5)
				MSE(×10^4^) 95% Cl	23 (20, 26)	30 (26,34)	41 (35,46)
				Coverage (%) 95% Cl	90.3 (87.5,93.1)	94.5 (92.6, 96.5)	95.3 (93.5, 97.1)
0	0	0.5	0.638	Bias(×10^3^) 95% Cl	- 53 (-56, -51)	- 31 (-34, -28)	2 (-2,7)
				MSE(×10^4^) 95% Cl	45 (40,49)	38 (33,43)	37 (40,53)
				Coverage (%) 95% Cl	72.5 (68.3,76.8)	91.4 (88.8, 93.9)	95.7 (93.9, 97.6)
0	0	1	0.76	Bias(×10^3^) 95% Cl	- 111 (-114, -108)	- 72 (-76, -67)	17 (11,24)
				MSE(×10^4^) 95% Cl	138 (131, 146)	83 (75,90)	67 (58,75)
				Coverage (%) 95% Cl	12.9 (10.3, 15.6)	66.1 (61.8, 70.5)	96.9 (95.2, 98.7)

* MW: Mann-Whitney estimate (i.e., *AUC_obs_*); R: Reiser's (2000) method; P: probit method.

** Simulation III was run 100 times. Each time, we generated 1000 simulated data sets. Each data set consists of 100 cases and 100 controls. each subject provides two replicate biomaker scores. Both true values and random errors were generated from log normal distributions with $\sigma_{X,\text{true}}^{2}=\sigma_{Y,\text{true}}^{2}=\sigma_{e_{X}}^{2}=\sigma_{e_{Y}}^{2}=1$.

**Table 4:** Estimate of *AUC_true_* and its 95% confidence interval for the NAPAP data.

	MW	R	P
*ÂUCtrue*95 % CI	0.589 [0.557,0.663]	0.611 [0.557,0.663]	0.618 [0.549,0.684]
