# Supplementary material for: Correcting AUC for Measurement Error
Source: J Biom Biostat. Author manuscript; Available in PMC 2017 Apr 28. (PMC5409172; doi:10.4172/2155-6180.1000270)
Supplement: Supplementary file [file NIHMS755928-supplement-Supplementary_file.pdf]

Online Supplementary Documents for "Correcting AUC for Measurement Error"

Bernard Rosner[1,2,*], Shelley Tworoger[1,3], and Weiliang Qiu[1]

[1]Channing Division of Network Medicine,

Brigham and Women's Hospital, Harvard Medical School,

181 Longwood Avenue, Boston, MA 02115, USA.

[2]Department of Biostatistics, Harvard T.H. Chan School of Public Health,

655 Huntington Avenue, Boston, Massachusetts 02115, USA.

[3]Department of Epidemiology, Harvard T.H. Chan School of Public Health,

677 Huntington Avenue Boston, Massachusetts 02115, USA.

December 17, 2015

A Derivation of AUC_{true} for diagnostic biomarkers measured without error

Define:

$$g(\mu) = \Phi^{-1}\left\{AUC(\mu)\right\}.$$

*Corresponding author. Email: stbar@channing.harvard.edu

The first order Taylor expansion is

$$g(\mu) \approx g(0) + g'(0)\mu,$$

where

$$\begin{aligned} g(0) =& \Phi^{-1}\left\{AUC(0)\right\} \\ g'(0) =& \left.\frac{dg(\mu)}{d\mu}\right|_{\mu=0}, \\ =& \left.\frac{d\Phi^{-1}\left\{AUC(\mu)\right\}}{d\mu}\right|_{\mu=0}. \end{aligned}$$

We can get

$$\begin{aligned} AUC(0) =& \int_{w=-\infty}^{\infty} \Phi(w)\phi(w)dw \\ \overset{\xi=\Phi(w)}{=}& \int_{\xi=0}^{1} \xi d\xi \\ =& \left.\frac{\xi^2}{2}\right|_{\xi=0}^{1} = \frac{1}{2}. \end{aligned}$$

Hence,

$$g(0) = \Phi^{-1}\left\{AUC(0)\right\} = \Phi^{-1}\left(\frac{1}{2}\right) = 0.$$

Based on

$$AUC\left(\mu\right) = \int_{w=-\infty}^{\infty} \Phi\left(w+\mu\right)\phi(w)dw,$$

we also can calculate

$$
\begin{aligned}
\left.\frac{dAUC(\mu)}{d\mu}\right|_{\mu=0} &= \int_{w=-\infty}^{\infty} \phi(w)\phi(w)dw \\
&= \int_{w=-\infty}^{\infty} \frac{1}{2\pi} \exp\left(-w^2\right) dw \\
&= \frac{\sqrt{1/2}}{\sqrt{2\pi}} \int_{w=-\infty}^{\infty} \frac{1}{\sqrt{2\pi}\sqrt{1/2}} \exp\left(-\frac{w^2}{2\frac{1}{2}}\right) dw \\
&= \frac{\sqrt{1/2}}{\sqrt{2\pi}} \\
&= \frac{1}{2\sqrt{\pi}}.
\end{aligned}
$$

From the definition of $g(\mu)$, we can get

$$
\begin{aligned}
\Phi\left\{g(\mu)\right\} &= AUC(\mu) \\
\phi\left\{g(\mu)\right\}\frac{dg(\mu)}{d\mu} &= \frac{dAUC(\mu)}{d\mu}.
\end{aligned}
$$

Hence,

$$
\begin{aligned}
\left.\frac{dg(\mu)}{d\mu}\right|_{\mu=0} &= \frac{1}{\phi\left\{g(0)\right\}} \left.\frac{dAUC(\mu)}{d\mu}\right|_{\mu=0} \\
&= \frac{1}{\phi(0)}\frac{1}{2\sqrt{\pi}} \\
&= \frac{1}{\frac{1}{\sqrt{2\pi}}}\frac{1}{2\sqrt{\pi}} \\
&= \frac{1}{\sqrt{2}}.
\end{aligned}
$$

Therefore,

$$g(\mu) \approx \frac{\mu}{\sqrt{2}}.$$

Hence,

$$AUC_{true}(\mu) \approx \Phi\left(\frac{\mu}{\sqrt{2}}\right). \tag{A1}$$

B Derivation of AUC_{true} for diagnostic biomarkers measured with error

The AUC is a function of μ:

$$
\begin{aligned}
AUC_{true}(\mu) =& Pr(Y < X) \\
=& \int_{x=-\infty}^{\infty} F_{Y,true}(x) f_X(x) dx \\
\overset{w=\Phi^{-1}(z)}{=}& \int_{w=-\infty}^{\infty} \Phi(w+\mu)\phi(w)dw.
\end{aligned}
\tag{A2}
$$

We have

$$AUC_{obs}(\mu)\,|e_y, e_x = \int_{x=-\infty}^{\infty} F_{Y,obs}(x) dF_{X,obs}(x).$$

Note that

$$\Phi^{-1}\{F_{Y,obs}(x)\} = H_{X,true}(x) + \mu + e_y.$$

That is,

$$F_{Y,obs}(x) = \Phi\{H_{X,true}(x) + \mu + e_y\}.$$

Hence,

$$AUC_{obs}(\mu)\,|e_y, e_x = \int_{x=-\infty}^{\infty} \Phi\{H_{X,true}(x) + \mu + e_y\} dF_{X,obs}(x).$$

Note that

$$
\begin{aligned}
F_{X,obs}(x) =& \Phi\{H_{X,true}(x) + e_x\}, \\
dF_{X,obs}(x) =& \phi\{H_{X,true}(x) + e_x\} dH_{X,true}(x).
\end{aligned}
$$

We have

$$AUC_{obs}(\mu)\,|e_y, e_x = \int_{x=-\infty}^{\infty} \Phi\{H_{X,true}(x) + \mu + e_y\} \phi\{H_{X,true}(x) + e_x\} dH_{X,true}(x)$$

Let $w = H_{X,true}(x) + e_x$. Then, based on Formulas (A1) and (A2), we have

$$\begin{aligned}
AUC_{obs}(\mu)\,|e_y, e_x &= \int_{x=-\infty}^{\infty} \Phi\left\{H_{X,true}(x) + \mu + e_y\right\} \phi\left\{H_{X,true}(x) + e_x\right\} dH_{X,true}(x) \\
&= \int_{w=-\infty}^{\infty} \Phi\left(w - e_x + \mu + e_y\right) \phi\left(w\right) dw \\
&= AUC\left(e_y - e_x + \mu\right) \\
&\approx \Phi\left(\frac{e_y - e_x + \mu}{\sqrt{2}}\right).
\end{aligned}$$

We have

$$\begin{aligned}
AUC_{obs}(\mu) &= \int_{e_y=-\infty}^{\infty}\int_{e_x=-\infty}^{\infty} AUC_{obs}\left(\mu|e_y, e_x\right) f_1\left(e_x\right) f_2\left(e_y\right) de_x de_y \\
&\approx \int_{e_y=-\infty}^{\infty}\int_{e_x=-\infty}^{\infty} \Phi\left(\frac{e_y - e_x + \mu}{\sqrt{2}}\right) \frac{1}{\sqrt{2\pi}\sigma_{e_y}} \exp\left(-\frac{e_y^2}{2\sigma_{e_y}^2}\right) \\
&\quad \frac{1}{\sqrt{2\pi}\sigma_{e_x}} \exp\left(-\frac{e_x^2}{2\sigma_{e_x}^2}\right) de_y de_x
\end{aligned}$$

Denote

$$\begin{aligned}
e_y^* &= \frac{e_y}{\sigma_{e_y}}, \\
e_x^* &= \frac{e_x}{\sigma_{e_x}}, \\
de_y &= de_y^* \sigma_{e_y}, \\
de_x &= de_x^* \sigma_{e_x}, \\
e_y^* &\sim \mathrm{N}\left(0, 1\right), \\
e_x^* &\sim \mathrm{N}\left(0, 1\right).
\end{aligned}$$

Then we have

$$AUC_{obs}(\mu) = \int_{e_y=-\infty}^{\infty}\int_{e_x=-\infty}^{\infty} \Phi\left(\frac{e_y^*\sigma_{e_y} - e_x^*\sigma_{e_x} + \mu}{\sqrt{2}}\right) \frac{1}{\sqrt{2\pi}} \exp\left(-\frac{e_y^{*2}}{2}\right) \frac{1}{\sqrt{2\pi}} \exp\left(-\frac{e_x^{*2}}{2}\right) de_y^* de_x^* \tag{A3}$$

Hence,

$$AUC_{obs}(0) = \int_{e_y=-\infty}^{\infty} \int_{e_x=-\infty}^{\infty} \Phi\left(\frac{e_y^* \sigma_{e_y} - e_x^* \sigma_{e_x}}{\sqrt{2}}\right) \frac{1}{\sqrt{2\pi}} \exp\left(-\frac{e_y^{*2}}{2}\right) \frac{1}{\sqrt{2\pi}} \exp\left(-\frac{e_x^{*2}}{2}\right) de_y^* de_x^*$$

$$=0.5.$$

The above equality can be derived as follows. For every $\left(e_x^*, e_y^*\right)$, there is a corresponding $\left(-e_x^*, -e_y^*\right)$ such that

$$g\left(e_x^*, e_y^*\right) = g\left(-e_x^*, -e_y^*\right),$$

where

$$g = \frac{1}{2\pi} \exp\left\{-\frac{1}{2}\left(e_x^{*2} + e_y^{*2}\right)\right\}.$$

Hence,

$$\begin{aligned}\int_{a=f\left(e_x^*, e_y^*\right)} \frac{\Phi(a) + \Phi(-a)}{2} da =& \frac{\Phi(a) + 1 - \Phi(a)}{2} \\ =& \Phi\left(\frac{e_y^* \sigma_{e_y} - e_x^* \sigma_{e_x}}{\sqrt{2}}\right) \\ =& 0.5.\end{aligned}$$

Denote

$$h(\mu) = \Phi^{-1}\left\{AUC_{obs}(\mu)\right\}.$$

Then we have

$$\begin{aligned} h(\mu) \approx& h(0) + \mu h'(\mu)|_{\mu=0} \\ h(0) =& \Phi^{-1}(0.5) = 0 \end{aligned} \tag{A4}$$

Now we calculate $h'(\mu)$.

$$\begin{aligned} AUC'_{obs}(\mu) =& \int_{e_y^*=-\infty}^{\infty} \int_{e_x^*=-\infty}^{\infty} \frac{1}{\sqrt{2}} \phi\left(\frac{e_y^* \sigma_{e_y} - e_x^* \sigma_{e_x} + \mu}{\sqrt{2}}\right) \frac{1}{2\pi} \exp\left\{-\frac{\left(e_y^{*2} + e_x^{*2}\right)}{2}\right\} de_y^* de_x^* \\ =& \int_{e_y^*=-\infty}^{\infty} \int_{e_x^*=-\infty}^{\infty} \frac{1}{\sqrt{2}} \frac{1}{\sqrt{2\pi}} \exp\left\{-\frac{1}{2} \frac{\left(e_y^* \sigma_{e_y} - e_x^* \sigma_{e_x} + \mu\right)^2}{2}\right\} \frac{1}{2\pi} \exp\left(-\frac{e_y^{*2} + e_x^{*2}}{2}\right) de_y^* de_x^* \end{aligned}$$

We have

$$AUC'_{obs}(0) = \int_{e_y^*=-\infty}^{\infty} \int_{e_x^*=-\infty}^{\infty} \frac{1}{\sqrt{2}} \frac{1}{\sqrt{2\pi}} \exp\left\{-\frac{1}{2}\frac{\left(e_y^*\sigma_{e_y} - e_x^*\sigma_{e_x}\right)^2}{2}\right\} \frac{1}{2\pi} \exp\left(-\frac{e_y^{*2}+e_x^{*2}}{2}\right) de_y^* de_x^* \tag{A5}$$

First integrate (A5) with respect to e_x^*.

$$\begin{aligned}
AUC'_{obs}(0|e_y^*) =& \int_{e_x^*=-\infty}^{\infty} \frac{1}{\sqrt{2}} \frac{1}{\sqrt{2\pi}} \exp\left\{-\frac{1}{2}\frac{\left(e_y^*\sigma_{e_y} - e_x^*\sigma_{e_x}\right)^2}{2}\right\} \frac{1}{\sqrt{2\pi}} \exp\left(-\frac{e_x^{*2}}{2}\right) de_x^* \\
=& \frac{1}{\sqrt{2}} \exp\left\{-\frac{1}{2}e_y^{*2}\left(\frac{\sigma_{e_y}^2}{2}\right)\right\} \frac{1}{\sqrt{2\pi}} \int_{e_x^*=-\infty}^{\infty} \frac{1}{\sqrt{2\pi}} \exp\left\{-\frac{1}{2}\left(\frac{e_x^{*2}\sigma_{e_x}^2 - 2e_y^*e_x^*\sigma_{e_y}\sigma_{e_x}}{2} + e_x^{*2}\right)\right\} de_x^* \\
=& \frac{1}{\sqrt{2}} \exp\left\{-\frac{1}{2}e_y^{*2}\left(\frac{\sigma_{e_y}^2}{2}\right)\right\} \frac{1}{\sqrt{2\pi}} \int_{e_x^*=-\infty}^{\infty} \frac{1}{\sqrt{2\pi}} \exp\left[-\frac{1}{2}\left\{e_x^{*2}\left(1+\frac{\sigma_{e_x}^2}{2}\right) - e_x^*e_y^*\sigma_{e_x}\sigma_{e_y}\right\}\right] de_x^*
\end{aligned}$$

Let

$$u = e_x^*\sqrt{1+\frac{\sigma_{e_x}^2}{2}}.$$

Then we have

$$\begin{aligned}
e_x^* =& \frac{u}{\sqrt{1+\frac{\sigma_{e_x}^2}{2}}} \\
de_x^* =& \frac{du}{\sqrt{1+\frac{\sigma_{e_x}^2}{2}}}
\end{aligned}$$

Then

$$\begin{aligned}
AUC'_{obs}(0|e_y^*) =& \frac{1}{\sqrt{2}} \exp\left\{-\frac{1}{2}e_y^{*2}\left(\frac{\sigma_{e_y}^2}{2}\right)\right\} \frac{1}{\sqrt{2\pi}} \int_{e_x^*=-\infty}^{\infty} \frac{1}{\sqrt{2\pi}} \exp\left\{-\frac{1}{2}\left(u^2 - \frac{u}{\sqrt{1+\frac{\sigma_{e_x}^2}{2}}}e_y^*\sigma_{e_x}\sigma_{e_y}\right)\right\} \frac{du}{\sqrt{1+\frac{\sigma_{e_x}^2}{2}}} \\
=& \frac{1}{\sqrt{2}} \exp\left\{-\frac{1}{2}e_y^{*2}\left(\frac{\sigma_{e_y}^2}{2}\right)\right\} \frac{1}{\sqrt{2\pi}} \frac{1}{\sqrt{1+\frac{\sigma_{e_x}^2}{2}}} \int_{e_x^*=-\infty}^{\infty} \frac{1}{\sqrt{2\pi}} \exp\left\{-\frac{1}{2}\left(u - \frac{e_y^*\sigma_{e_x}\sigma_{e_y}}{2\sqrt{1+\frac{\sigma_{e_x}^2}{2}}}\right)^2\right\} \\
& \cdot du \exp\left\{\frac{1}{2}\left(\frac{e_y^*\sigma_{e_x}\sigma_{e_y}}{2\sqrt{1+\frac{\sigma_{e_x}^2}{2}}}\right)^2\right\}
\end{aligned}$$

Thus

$$AUC'_{obs}(0|e_y^*) = \frac{1}{\sqrt{2}} \exp\left\{ -\frac{1}{2} e_y^{*2} \left(\frac{\sigma_{e_y}^2}{2} \right) \right\} \frac{1}{\sqrt{1+\frac{\sigma_{e_x}^2}{2}}} \frac{1}{\sqrt{2\pi}} \exp\left\{ \frac{1}{2} \left(\frac{e_y^* \sigma_{e_x} \sigma_{e_y}}{2\sqrt{1+\frac{\sigma_{e_x}^2}{2}}} \right)^2 \right\}$$

Furthermore,

$$\begin{aligned} AUC'_{obs}(0) &= \int_{e_y^*=-\infty}^{\infty} AUC'_{obs}(0|e_y^*) f(e_y^*) de_y^* \\ &= \frac{1}{\sqrt{2}} \frac{1}{\sqrt{2\pi}} \frac{1}{\sqrt{1+\frac{\sigma_{e_x}^2}{2}}} \int_{e_y^*=-\infty}^{\infty} \exp\left\{ -\frac{1}{2} \left(e_y^{*2} \frac{\sigma_{e_y}^2}{2} \right) \right\} \exp\left\{ \frac{1}{2} \left(\frac{e_y^* \sigma_{e_y} \sigma_{e_x}}{2\sqrt{1+\frac{\sigma_{e_x}^2}{2}}} \right)^2 \right\} \frac{1}{\sqrt{2\pi}} \exp\left(-\frac{1}{2} e_y^{*2} \right) de_y^* \\ &= \frac{1}{\sqrt{2}} \frac{1}{\sqrt{2\pi}} \frac{1}{\sqrt{1+\frac{\sigma_{e_x}^2}{2}}} \int_{e_y^*=-\infty}^{\infty} \exp\left[-\frac{1}{2} e_y^{*2} \left\{ \frac{\sigma_{e_y}^2}{2} + 1 - \frac{\left(\sigma_{e_y}\sigma_{e_x}\right)^2}{\left(2\sqrt{1+\frac{\sigma_{e_x}^2}{2}}\right)^2} \right\} \right] \frac{1}{\sqrt{2\pi}} de_y^* \end{aligned}$$

Let

$$v = e_y^* \sqrt{1 + \frac{\sigma_{e_y}^2}{2} - \frac{\sigma_{e_y}^2 \sigma_{e_x}^2}{4\left(1+\frac{\sigma_{e_x}^2}{2}\right)}}$$

Then we have

$$dv = de_y^* \sqrt{1 + \frac{\sigma_{e_y}^2}{2} - \frac{\sigma_{e_y}^2 \sigma_{e_x}^2}{4\left(1+\frac{\sigma_{e_x}^2}{2}\right)}}$$

Thus

$$\begin{aligned} AUC'_{obs}(0) &= \frac{1}{\sqrt{2}} \frac{1}{\sqrt{2\pi}} \frac{1}{\sqrt{1+\frac{\sigma_{e_x}^2}{2}}} \int_{v=-\infty}^{\infty} \exp\left(-\frac{1}{2} v^2 \right) \frac{1}{\sqrt{2\pi}} \frac{dv}{\sqrt{1 + \frac{\sigma_{e_y}^2}{2} - \frac{\sigma_{e_y}^2 \sigma_{e_x}^2}{4\left(1+\frac{\sigma_{e_x}^2}{2}\right)}}} \\ &= \frac{1}{\sqrt{2}} \frac{1}{\sqrt{2\pi}} \frac{1}{\sqrt{1+\frac{\sigma_{e_x}^2}{2}}} \frac{1}{\sqrt{1 + \frac{\sigma_{e_y}^2}{2} - \frac{\sigma_{e_y}^2 \sigma_{e_x}^2}{4\left(1+\frac{\sigma_{e_x}^2}{2}\right)}}} \end{aligned}$$

Thus, because $h'(0) = \sqrt{2\pi} AUC'(0)$, it follows that

$$
\begin{aligned}
h'(0) =& \frac{1}{\sqrt{2}} \frac{1}{\sqrt{1 + \frac{\sigma_{e_x}^2}{2}}} \frac{1}{\sqrt{1 + \frac{\sigma_{e_y}^2}{2} - \frac{\sigma_{e_y}^2 \sigma_{e_x}^2}{4\left(1 + \frac{\sigma_{e_x}^2}{2}\right)}}} \\
=& \frac{1}{\sqrt{\left(1 + \frac{\sigma_{e_x}^2 + \sigma_{e_y}^2}{2}\right)}} \frac{1}{\sqrt{2}}
\end{aligned}
\tag{A6}
$$

Thus, from (A3), (A4), and (A6), we have

$$
h(\mu) = \Phi^{-1}\left(AUC_{obs}\left(\mu\right)\right) \approx 0 + \frac{\mu}{\sqrt{\left(1 + \frac{\sigma_{e_x}^2 + \sigma_{e_y}^2}{2}\right)}} \frac{1}{\sqrt{2}},
$$

or

$$
AUC_{obs}(\mu) \approx \Phi \left\{ \frac{\mu / \sqrt{2}}{\sqrt{\left(1 + \frac{\sigma_{e_x}^2 + \sigma_{e_y}^2}{2}\right)}} \right\}
$$

Hence, based on (A1), we have

$$
AUC_{obs}(\mu) \approx \Phi \left[\frac{\Phi^{-1}\left\{AUC_{true}(\mu)\right\}}{\sqrt{\left(1 + \frac{\sigma_{e_x}^2 + \sigma_{e_y}^2}{2}\right)}} \right] \tag{A7}
$$

Thus, if $\sigma_{e_y}^2 = \sigma_{e_x}^2 = 0$, then

$$
AUC_{obs}(\mu) = \Phi\left[\Phi^{-1}\left\{AUC_{true}(\mu)\right\}\right] = AUC_{true}\left(\mu\right).
$$

In general,

$$
AUC_{obs}(\mu) < AUC_{true}(\mu)
$$

Equivalently, from (A7) we have

$$AUC_{true}(\mu) = \Phi\left[\Phi^{-1}\left\{AUC_{obs}(\mu)\right\}\sqrt{\left(1+\frac{\sigma_{e_x}^2+\sigma_{e_y}^2}{2}\right)}\right] \tag{A8}$$

This is the measurement error correction for AUC.

If $\sigma_{e_x}^2 = \sigma_{e_y}^2 = \sigma_e^2$, then

$$AUC_{true}(\mu) = \Phi\left[\Phi^{-1}\left\{AUC_{obs}(\mu)\right\}\sqrt{(1+\sigma_e^2)}\right]$$

We can estimate $\sigma_{e_x}^2$ and $\sigma_{e_y}^2$ from repeated measures of the probit score on the same subjects and substitute into the above equation (A8).

Recall our measurement error model (10), if $V_x = H_{x,obs}$ and $V_y = H_{y,obs}$, then

$$\begin{aligned} V_x =& H_x + e_x, \quad \text{Var}(H_x) = 1, \\ V_y =& H_y + e_y, \quad \text{Var}(H_y) = 1. \end{aligned}$$

Hence, we have

$$\begin{aligned} \text{Var}(V_x) =& 1 + \text{Var}(e_x), \\ \text{Var}(V_y) =& 1 + \text{Var}(e_y). \end{aligned}$$

Denote $\sigma_{V_x} = \text{Var}(V_x)$ and $\sigma_{V_y} = \text{Var}(V_y)$. Then we have

$$\begin{aligned} \sigma_{V_x}^2 =& 1 + \sigma_{e_x}^2, \\ \sigma_{V_y}^2 =& 1 + \sigma_{e_y}^2 \end{aligned}$$

Hence, intra-class correlations are

$$
\begin{aligned}
ICC_x =& \frac{\sigma^2_{H_x}}{\sigma^2_{V_x}} = \frac{1}{1+\sigma^2_{e_x}}, \\
ICC_y =& \frac{\sigma^2_{H_y}}{\sigma^2_{V_y}} = \frac{1}{1+\sigma^2_{e_y}}.
\end{aligned}
$$

$$
\begin{aligned}
\sigma^2_{e_x} =& \frac{1}{ICC_x} - 1, \\
\sigma^2_{e_y} =& \frac{1}{ICC_y} - 1.
\end{aligned}
$$

Thus, (A8) can be rewritten in the form:

$$
AUC_{true}(\mu) = \Phi\left[\Phi^{-1}\left\{AUC_{obs}(\mu)\right\} \times \sqrt{\frac{\frac{1}{ICC_x} + \frac{1}{ICC_y}}{2}}\right], \tag{A9}
$$

where agree with Equation 7 in the main text.

If $ICC_x = ICC_y = ICC$, then

$$
AUC_{true}(\mu) = \Phi\left[\Phi^{-1}\left\{AUC_{obs}(\mu)\right\} / \sqrt{ICC}\right].
$$

If $ICC = 1$, then $AUC_{true}(\mu) = AUC_{obs}(\mu)$.

If $ICC = 0$, then $AUC_{true}(\mu) = \Phi(\infty) = 1$.

C Confidence Limits for $AUC_{\textbf{corrected}}$

$$
AUC_{true}(\mu) = \Phi\left[\Phi^{-1}\left\{AUC_{obs}(\mu)\right\} \times \sqrt{\frac{\frac{1}{ICC_x} + \frac{1}{ICC_y}}{2}}\right].
$$

where ICC_x and ICC_y are intra-class correlations

$$\begin{aligned} ICC_x =& \frac{1}{1+\sigma_{e_x}^2}, \\ ICC_y =& \frac{1}{1+\sigma_{e_y}^2}. \end{aligned}$$

We assume there exists at least one replicated observation for each subject in the data set or in a subset of the data set so that we can estimate intra-class correlations.

We can use the Mann-Whitney U statistic to estimate $AUC_{obs}(\mu)$ (c.f. Formula A13 in Section D.1).

We use the delta method to derive the variance of the corrected AUC.

Denote

$$a =\Phi^{-1}\left\{AUC_{obs}\left(\mu\right)\right\}, \quad b = \sqrt{\frac{\frac{1}{ICC_x}+\frac{1}{ICC_y}}{2}}.$$

We have

$$AUC_{true} = \Phi\left\{\Phi^{-1}\left[AUC_{obs}\left(\mu\right)\right]\sqrt{\frac{\frac{1}{ICC_x}+\frac{1}{ICC_y}}{2}}\right\} = \Phi\left(a\times b\right) \tag{A10}$$

An approximate $100\% \times (1-\alpha)$ CI for AUC_{true} is given by $[\Phi(c_1), \Phi(c_2)]$, where

$$(c_1, c_2) = \left[\left(\hat{a}\times\hat{b}\right) - z_{1-\alpha/2}se\left(\hat{a}\times\hat{b}\right), \left(\hat{a}\times\hat{b}\right) + z_{1-\alpha/2}se\left(\hat{a}\times\hat{b}\right)\right],$$

where

$$\begin{aligned} \hat{a} =& \Phi^{-1}\left\{A\hat{U}C_{obs}\left(\hat{\mu}\right)\right\} \\ \hat{b} =& \sqrt{\frac{\frac{1}{I\hat{C}C_x}+\frac{1}{I\hat{C}C_y}}{2}} \end{aligned}$$

Denote

$$g\left(\hat{a},\hat{b}\right) = \hat{a}\times\hat{b}.$$

By Taylor expansion, we have

$$g\left(\hat{a},\hat{b}\right) \approx g\left(a,b\right) + \left.\frac{\partial g\left(\hat{a},\hat{b}\right)}{\partial \hat{a}}\right|_{\hat{a}=a,\hat{b}=b}\left(\hat{a}-a\right) + \left.\frac{\partial g\left(\hat{a},\hat{b}\right)}{\partial \hat{b}}\right|_{\hat{a}=a,\hat{b}=b}\left(\hat{b}-b\right)$$

Assuming $\hat{a}$ and $\hat{b}$ are independent, we then can approximate the variance of $g\left(\hat{a},\hat{b}\right)$ by

$$\text{Var}\left\{g\left(\hat{a},\hat{b}\right)\right\} \approx \left\{\left.\frac{\partial g\left(\hat{a},\hat{b}\right)}{\partial \hat{a}}\right|_{\hat{a}=a,\hat{b}=b}\right\}^2 \text{Var}\left(\hat{a}\right) + \left\{\left.\frac{\partial g\left(\hat{a},\hat{b}\right)}{\partial \hat{b}}\right|_{\hat{a}=a,\hat{b}=b}\right\}^2 \text{Var}\left(\hat{b}\right)$$

We have

$$\left.\frac{\partial g\left(\hat{a},\hat{b}\right)}{\partial a}\right|_{\hat{a}=a,\hat{b}=ICC_y} = b,$$
$$\left.\frac{\partial g\left(\hat{a},\hat{b}\right)}{\partial ICC_y}\right|_{\hat{a}=a,\hat{b}=ICC_y} = a.$$

Thus,

$$\text{Var}\left\{g\left(\hat{a},\hat{b}\right)\right\} \approx \hat{b}^2\text{Var}\left(\hat{a}\right) + \hat{a}^2\text{Var}\left(\hat{b}\right) \tag{A11}$$

and

$$se\left(\hat{a}\times\hat{b}\right) = \sqrt{\left\{\frac{\frac{1}{ICC_x}+\frac{1}{ICC_y}}{2}\right\}\text{Var}\left(\hat{a}\right) + \left[\Phi^{-1}\left\{AUC_{obs}\left(\mu\right)\right\}\right]^2\text{Var}\left(\hat{b}\right)}$$

D Delta method to approximate $\text{Var}\left(\hat{a}\right)$, $\text{Var}\left(\hat{b}\right)$

D.1 Delta method to approximate $\text{Var}\left(\hat{a}\right)$

Denote

$$\begin{aligned} g_2\left(AUC\right) =& \Phi^{-1}\left(AUC\right) \\ AUC =& AUC_{obs}(\mu) \end{aligned}$$

Then

$$\hat{a} = g_2\left(A\hat{U}C\right)$$

By the delta method, the approximate variance of $\hat{a}$ is

$$\text{Var}\left(\hat{a}\right) = \left\{ \left. \frac{dg_2\left(A\hat{U}C\right)}{dA\hat{U}C} \right|_{A\hat{U}C=AUC} \right\}^2 \text{Var}\left(A\hat{U}C\right)$$

Note that

$$A\hat{U}C = \Phi\left\{g_2\left(A\hat{U}C\right)\right\}.$$

If we take derivative on both sides, we obtain

$$\begin{aligned} 1 =& \frac{d\Phi\left\{g_2\left(A\hat{U}C\right)\right\}}{dA\hat{U}C} \\ =& \phi\left\{g_2\left(A\hat{U}C\right)\right\}\frac{dg_2\left(A\hat{U}C\right)}{dA\hat{U}C}. \end{aligned}$$

Hence, we have

$$\begin{aligned} \frac{dg_2\left(A\hat{U}C\right)}{dA\hat{U}C} =& \frac{1}{\phi\left\{g_2\left(A\hat{U}C\right)\right\}} \\ =& \frac{1}{\phi\left\{\Phi^{-1}\left(A\hat{U}C\right)\right\}} \end{aligned}$$

Therefore

$$\begin{aligned}\text{Var}\ (\hat{a}) =& \text{Var}\ \left\{\Phi^{-1}\left(A\hat{U}C\right)\right\} \\ \approx& \left[\frac{1}{\phi\left\{\Phi^{-1}\left(AUC\right)\right\}}\right]^2 \text{Var}\ \left(A\hat{U}C\right) \\ =& \frac{\text{Var}\ \left(A\hat{U}C\right)}{\left[\phi\left\{\Phi^{-1}\left(A\hat{U}C\right)\right\}\right]^2}\end{aligned} \tag{A12}$$

Based on the relationship between AUC and the Mann-Whitney statistic

$$\begin{aligned}A\hat{U}C =& \hat{P}r\left(Y < X\right) \\ =& \frac{U}{n_X n_Y}\end{aligned} \tag{A13}$$

where n_X and n_Y are the sample sizes for the X and Y samples, respectively, and

$$U = \sum_{i,j} I\left(Y_j < X_i\right)$$

$$I\left(Y_j < X_i\right) = \begin{cases} 1 & \text{if } Y_j < X_i \\ 1/2 & \text{if } Y_j = X_i \\ 0 & \text{if } Y_j > X_i \end{cases}$$

Denote

$$\hat{\theta} = \frac{U}{n_X n_Y}.$$

That is,

$$\hat{\theta} = A\hat{U}C.$$

It is well-known that under $H_0 : F_X = F_Y$, where F_X and F_Y are cumulative distribution

functions of the random variables X and Y, respectively,

$$\begin{aligned}\text{Var}\left(A\hat{U}C\right) =& \text{Var}\left(\hat{\theta}\right)\\ \overset{H_0}{=}& \frac{(n_X+n_Y+1)}{12n_Xn_Y}\end{aligned}$$

Under the probit-shift model

$$F_Y(y)=\Phi\left[\Phi^{-1}\left\{F_X(y)\right\}+\mu\right],$$

Rosner and Glynn (2009) [1] derived the approximate variance of $\hat{\theta}$ under H_1

$$\begin{aligned}\text{Var}\left(A\hat{U}C\right) =& \text{Var}\left(\hat{\theta}\right)\\ =& \{\theta\,(1-\theta)+(n_X+n_Y-2)\\ \times& \left[\Phi_2\left\{\Phi^{-1}\left(\theta\right),\Phi^{-1}\left(\theta\right),1/2\right\}-\theta^2\right]\}/\left(n_Xn_Y\right)\end{aligned}$$

where

$$\Phi_2\left(x,y,\rho\right)=Pr\left[Z_1<x\text{ and }Z_2<y\,\middle|\,(Z_1,Z_2)^T\sim\text{N}\left\{\begin{pmatrix}0\\0\end{pmatrix},\begin{pmatrix}1&\rho\\ \rho&1\end{pmatrix}\right\}\right],$$

which was used to estimate $\text{Var}\left(A\hat{U}C\right)$ in (A12).

D.2 Delta method to approximate $\text{Var}\left(\hat{b}\right)$

Denote

$$h\left(I\hat{C}C_x,I\hat{C}C_y\right)=\sqrt{\frac{\frac{1}{I\hat{C}C_x}+\frac{1}{I\hat{C}C_y}}{2}}$$

By Taylor expansion, we have

$$h\left(I\hat{C}C_x, I\hat{C}C_y\right) \approx h\left(ICC_x, ICC_y\right) + \left.\frac{\partial h\left(I\hat{C}C_x, I\hat{C}C_y\right)}{\partial I\hat{C}C_x}\right|_{I\hat{C}C_x=ICC_x, I\hat{C}C_y=ICC_y} \left(I\hat{C}C_x - ICC_x\right) + \left.\frac{\partial h\left(I\hat{C}C_x, I\hat{C}C_y\right)}{\partial I\hat{C}C_y}\right|_{I\hat{C}C_x=ICC_x, I\hat{C}C_y=ICC_y} \left(I\hat{C}C_y - ICC_y\right)$$

Assuming $I\hat{C}C_x$ and $I\hat{C}C_y$ are independent, we then can approximate the variance of $h\left(I\hat{C}C_x, I\hat{C}C_y\right)$ by

$$\mathrm{Var}\left\{h\left(I\hat{C}C_x, I\hat{C}C_y\right)\right\} \approx \left\{\left.\frac{\partial h\left(I\hat{C}C_x, I\hat{C}C_y\right)}{\partial I\hat{C}C_x}\right|_{I\hat{C}C_x=ICC_x, I\hat{C}C_y=ICC_y}\right\}^2 \mathrm{Var}\left(I\hat{C}C_x\right) + \left\{\left.\frac{\partial h\left(I\hat{C}C_y, I\hat{C}C_y\right)}{\partial I\hat{C}C_y}\right|_{I\hat{C}C_x=ICC_x, I\hat{C}C_y=ICC_y}\right\}^2 \mathrm{Var}\left(I\hat{C}C_y\right)$$

We have

$$\left.\frac{\partial h\left(I\hat{C}C_x, I\hat{C}C_y\right)}{\partial ICC_x}\right|_{I\hat{C}C_x=ICC_x, I\hat{C}C_y=ICC_y} = -\frac{1}{\left(2\sqrt{2}\right) ICC_x^2 \sqrt{\frac{1}{ICC_x} + \frac{1}{ICC_y}}}$$

$$\left.\frac{\partial h\left(I\hat{C}C_x, I\hat{C}C_y\right)}{\partial ICC_y}\right|_{I\hat{C}C_x=ICC_x, I\hat{C}C_y=ICC_y} = -\frac{1}{\left(2\sqrt{2}\right) ICC_y^2 \sqrt{\frac{1}{ICC_x} + \frac{1}{ICC_y}}}$$

The approximate variance of sample intra-class correlation r_I is [2]

$$\mathrm{Var}\left(r_I\right) = \frac{2\left(1 - r_I\right)^2 \left\{1 + \left(k_0 - 1\right) r_I\right\}^2}{\left\{k_0 \left(k_0 - 1\right)\left(n_1 - 1\right)\right\}}$$

where n_1 is the number of subjects, k_i is the number of replicates available for the i-th

subject, and

$$k_0 = \frac{1}{n_1 - 1}\left(\sum_{i=1}^{n_1} k_i - \frac{\sum_{i=1}^{n_1} k_i^2}{\sum_{i=1}^{n_1} k_i}\right)$$

In our example, we estimated r_I separately for cases and controls. Hence, $n_1 = n_X$ for cases and $n_1 = n_Y$ for controls.

If $k_i = k$, $i = 1, \ldots, n_1$, then $k_0 = k$. Thus,

$$\begin{aligned}\text{Var}\left(\hat{b}\right) =& \frac{\text{Var}\left(\widehat{ICC_x}\right)}{8\left(ICC_x\right)^4\left[\frac{1}{ICC_x}+\frac{1}{ICC_y}\right]} + \frac{\text{Var}\left(\widehat{ICC_y}\right)}{8\left(ICC_y\right)^4\left[\frac{1}{ICC_x}+\frac{1}{ICC_y}\right]} \\ =& \frac{1}{8\left(\frac{1}{ICC_x}+\frac{1}{ICC_y}\right)}\left\{\frac{\text{Var}\left(\widehat{ICC_x}\right)}{\widehat{ICC}_x^4} + \frac{\text{Var}\left(\widehat{ICC_y}\right)}{\widehat{ICC}_y^4}\right\}.\end{aligned} \tag{A14}$$

Upon substitution of (A12) and (A14), we obtain $se\left(\hat{a} \times \hat{b}\right)$ in (A11) and a $100\% \times (1-\alpha)$ CI for AUC_{true} in (A10) and (A8).

E True AUC for Simulation II and Simulation III

We have from (9) and (10) that

$$\begin{aligned}AUC_{true} =& Pr\left(Y_{true} < X_{true}\right) \\ =& Pr\left\{\log(Y_{true}) < \log(X_{true})\right\} \\ =& Pr\left\{\log(Y_{true}) - \log(X_{true}) < 0\right\}.\end{aligned}$$

Since $\log(Y_{true})$ and $\log(X_{true})$ are independent, we have

$$\log(Y_{true}) - \log(X_{true}) \sim \text{N}\left(-\mu, \sigma^2_{X,true} + \sigma^2_{Y,true}\right)$$

Hence,

$$\frac{\{\log(Y_{true}) - \log(X_{true})\} + \mu}{\sqrt{\sigma^2_{X,true} + \sigma^2_{Y,true}}} \sim \mathrm{N}(0,1).$$

Therefore,

$$\begin{aligned} AUC_{true} =& Pr\left(Y_{true} < X_{true}\right) \\ =& Pr\left\{\log(Y_{true}) - \log(X_{true}) < 0\right\} \\ =& Pr\left\{\frac{\log(Y_{true}) - \log(X_{true}) + \mu}{\sqrt{\sigma^2_{X,true} + \sigma^2_{Y,true}}} < \frac{\mu}{\sqrt{\sigma^2_{X,true} + \sigma^2_{Y,true}}}\right\} \\ =& \Phi\left(\frac{\mu}{\sqrt{\sigma^2_{X,true} + \sigma^2_{Y,true}}}\right) \end{aligned}$$

F Simulation results

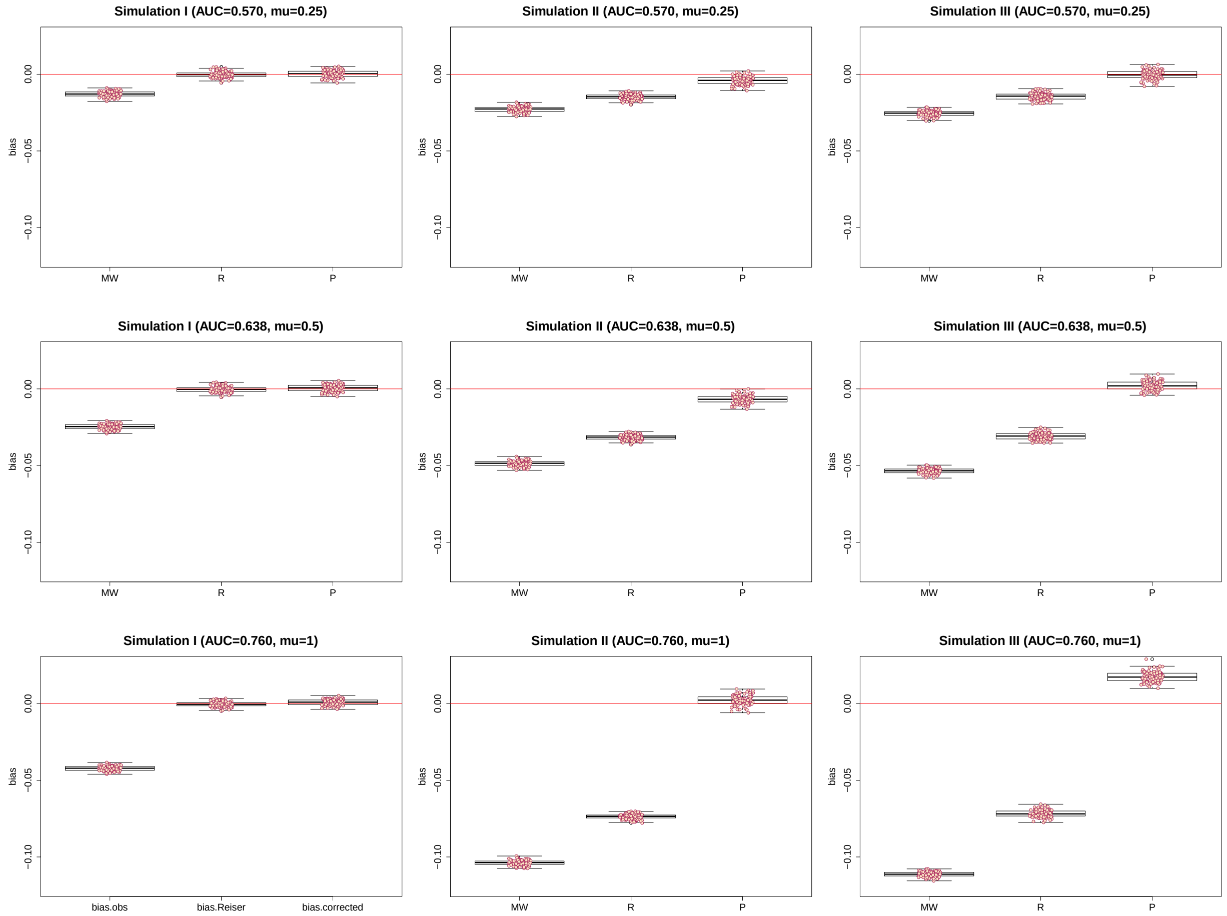

Figure S1: Parallel boxplots of bias. The upper panel: $\mu = 0.25$; The middle panel: $\mu = 0.5$; The bottom panel: $\mu = 1$; The left column: Simulation I; The middle column: Simulation II; The right column: Simulation III.

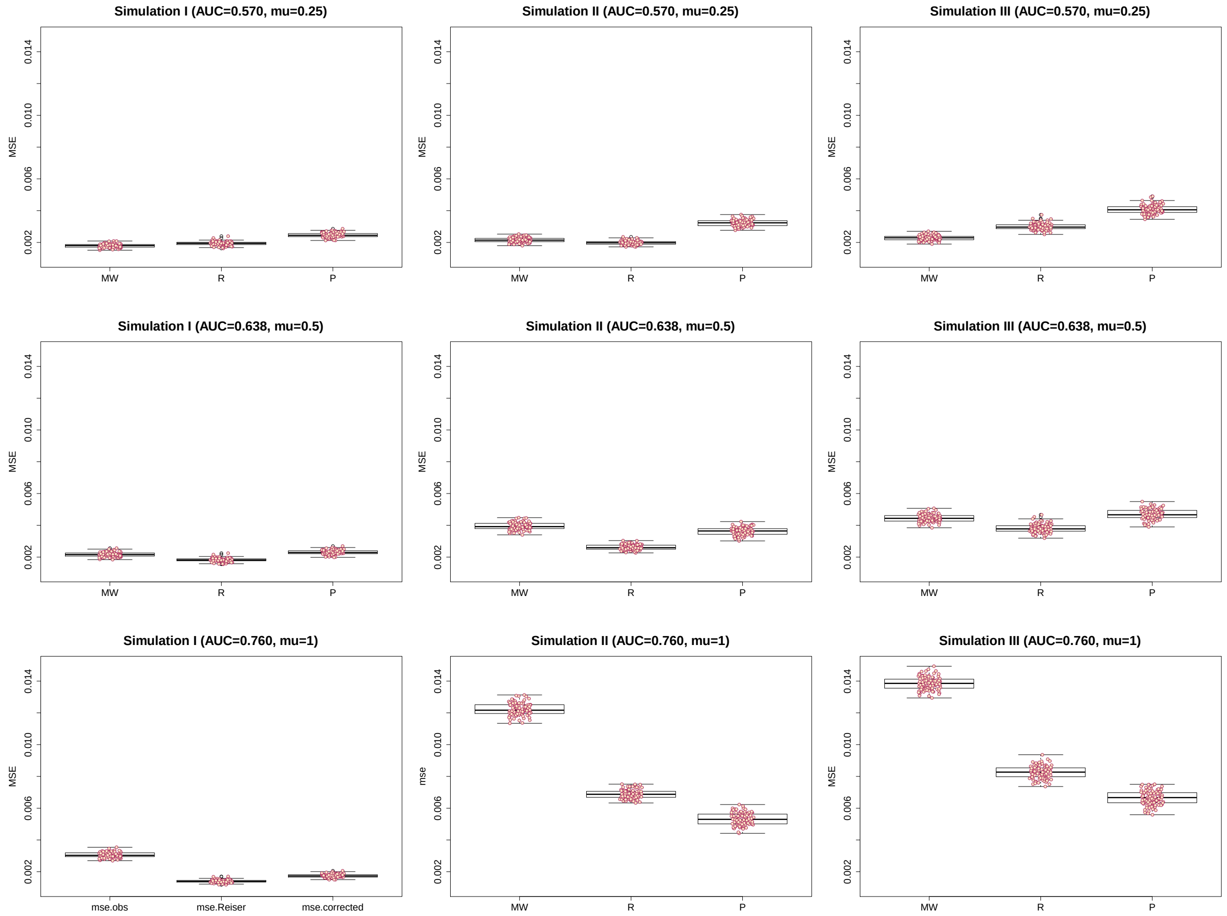

Figure S2: Parallel boxplots of MSE. The upper panel: $\mu = 0.25$; The middle panel: $\mu = 0.5$; The bottom panel: $\mu = 1$; The left column: Simulation I; The middle column: Simulation II; The right column: Simulation III.

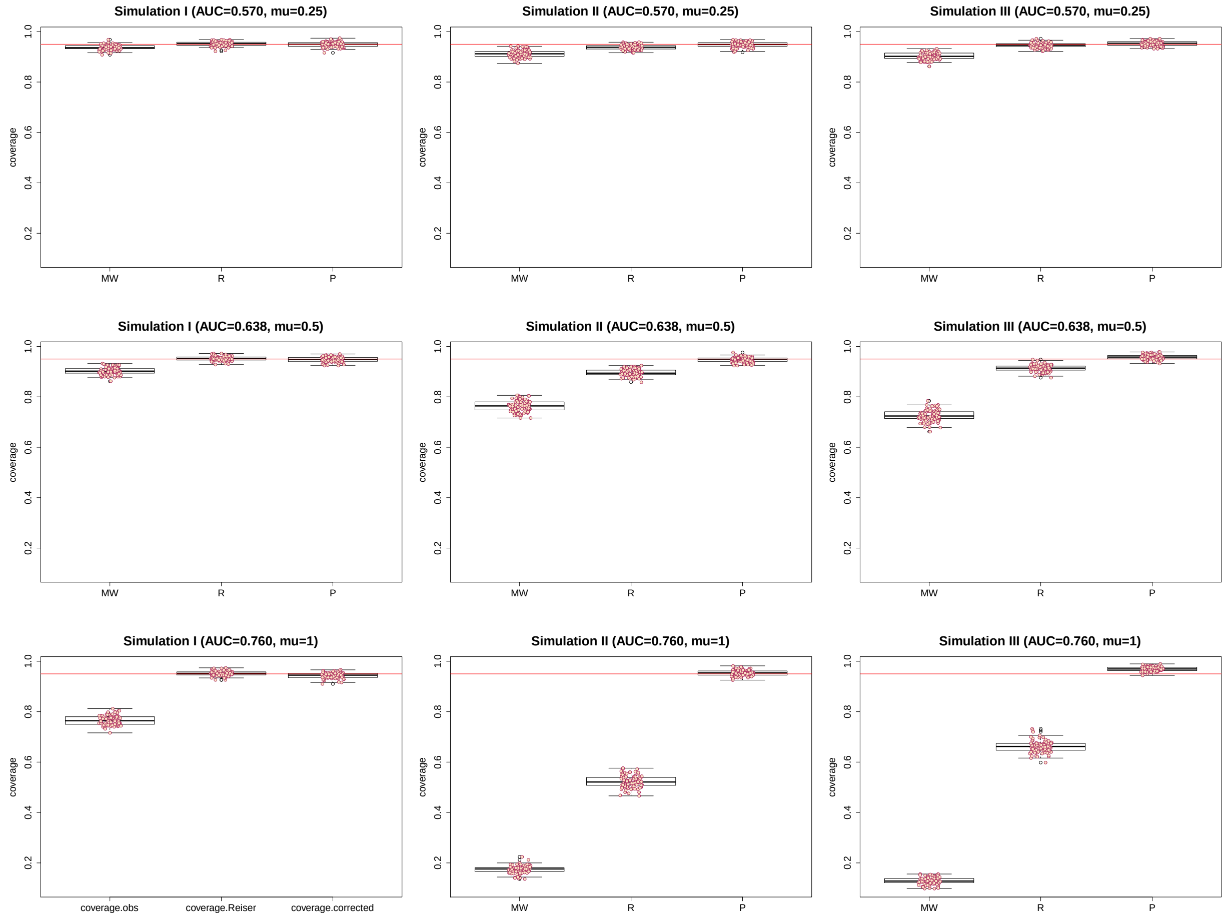

Figure S3: Parallel boxplots of coverage. The upper panel: $\mu = 0.25$; The middle panel: $\mu = 0.5$; The bottom panel: $\mu = 1$; The left column: Simulation I; The middle column: Simulation II; The right column: Simulation III.

References

[1] Rosner, B. and Glynn, R.J. Power and sample size estimation for the Wilcoxon rank sum test with application to comparisons of c statistics from alternative prediction models. *Biometrics*, 65:188–197, 2009.

[2] Donner, A. A review of inference procedures for the intraclass correlation coefficient in the one-way random effects model. *International Statistical Review*, 54(1):67–82, 1986.

Table S1: Bias, Mean Square Error (MSE), and Coverage for $AUC_{true}(\mu)$ from Simulation I** with 50 subjects per group, $\sigma_X^2/\sigma_Y^2 = 2$, and $\theta^2 = 1/3$

λ	μ_Y	μ_X	AUC_{true}		MW*	R*	P*
0	0	0.25	0.570	Bias($\times 10^3$)	-8	0	1
				95%CI	$(-13, -3)$	$(-5, 4)$	$(-4, 7)$
				MSE($\times 10^4$)	34	37	47
				95%CI	$(30, 38)$	$(32, 41)$	$(41, 53)$
				Coverage(%)	94.3	95.1	94.6
				95%CI	$(92.2, 96.4)$	$(93.2, 97.0)$	$(92.5, 96.7)$
0	0	0.50	0.638	Bias($\times 10^3$)	-15	0	2
				95%CI	$(-20, -11)$	$(-5, 4)$	$(-3, 8)$
				MSE($\times 10^4$)	34	35	45
				95%CI	$(30, 39)$	$(31, 40)$	$(39, 50)$
				Coverage (%)	93.6	95.2	94.6
				95%CI	$(91.6, 95.6)$	$(93.3, 97.1)$	$(92.5, 96.7)$
0	0	1.0	0.760	Bias($\times 10^3$)	-27	0	4
				95%CI	$(-31, -23)$	$(-5, 4)$	$(0, 9)$
				MSE($\times 10^4$)	35	29	37
				95%CI	$(31, 39)$	$(26, 33)$	$(32, 42)$
				Coverage (%)	91.3	95.1	94.3
				95%CI	$(89.2, 93.5)$	$(93.1, 97.1)$	$(92.1, 96.4)$

* MW: Mann-Whitney estimate (i.e., AUC_{obs}); R: Reiser's (2000) method; P: probit method.

** Simulation I was run 100 times. Each time, we generated 1000 simulated data sets. Each data set consists of 50 cases and 50 controls. Each subject provides two replicate biomaker scores. Both true values and random errors are assumed to come from normal distributions with $\sigma_x^2 = 2$, $\sigma_y^2 = 1$, $\sigma_\epsilon^2 = \sigma_\eta^2 = 0.5$.

Table S2: Bias, Mean Square Error (MSE), and Coverage for $AUC_{true}(\mu)$ from Simulation II** with 50 subjects per group, $\sigma_X^2/\sigma_Y^2 = 2$, and $\theta^2 = 2/3$.

λ	μ_Y	μ_X	AUC_{true}		MW*	R*	P*
0	0	0.25	0.570	Bias($\times 10^3$)	-12	-15	-2
				95%CI	$(-17, -7)$	$(-20, -10)$	$(-9, 4)$
				MSE($\times 10^4$)	35	34	53
				95%CI	$(31, 39)$	$(31, 38)$	$(47, 59)$
				Coverage(%)	94.1	94.8	94.8
				95%CI	$(91.9, 96.3)$	$(92.9, 96.7)$	$(92.7, 96.8)$
0	0	0.50	0.638	Bias($\times 10^3$)	-8	15	42
				95%CI	$(-13, -3)$	$(11, 18)$	$(34, 49)$
				MSE($\times 10^4$)	32	18	93
				95%CI	$(29, 36)$	$(15, 20)$	$(79, 108)$
				Coverage (%)	94.3	99.0	94.8
				95%CI	$(92.3, 96.3)$	$(98.2, 99.8)$	$(93.0, 96.7)$
0	0	1.0	0.760	Bias($\times 10^3$)	-52	-51	57
				95%CI	$(-57, -48)$	$(-54, -47)$	$(49, 65)$
				MSE($\times 10^4$)	57	42	126
				95%CI	$(51, 63)$	$(38, 46)$	$(112, 141)$
				Coverage (%)	82.3	90.2	98.1
				95%CI	$(79.1, 85.5)$	$(87.4, 93.0)$	$(96.9, 99.3)$

* MW: Mann-Whitney estimate (i.e., AUC_{obs}); R: Reiser's (2000) method; P: probit method.

** Simulation II was run 100 times. Each time, we generated 1000 simulated data sets. Each data set consists of 50 cases and 50 controls. Each subject provides two replicate biomaker scores. True values were generated from log normal distributions and random errors were generated from normal distributions with $\sigma_X^2 = 2$, $\sigma_Y^2 = 1$, $\sigma_{e_X}^2 = \sigma_{e_Y}^2 = 1$.

Table S3: Bias, Mean Square Error (MSE), and Coverage for $AUC_{true}\,(\mu)$ from Simulation III** with 50 subjects per group, $\sigma_X^2/\sigma_Y^2 = 2$, and $\theta^2 = 2/3$.

λ	μ_Y	μ_X	AUC_{true}		MW*	R*	P*
0	0	0.25	0.570	Bias($\times 10^3$)	6	43	38
				95%CI	$(1, 11)$	$(39, 48)$	$(30, 45)$
				MSE($\times 10^4$)	33	44	94
				95%CI	$(29, 37)$	$(39, 50)$	$(80, 107)$
				Coverage(%)	94.4	95.7	94.3
				95%CI	$(92.5, 96.4)$	$(93.8, 97.6)$	$(92.4, 96.1)$
0	0	0.50	0.638	Bias($\times 10^3$)	-15	15	45
				95%CI	$(-19, -10)$	$(11, 19)$	$(37, 53)$
				MSE($\times 10^4$)	34	26	112
				95%CI	$(30, 38)$	$(21, 30)$	$(95, 128)$
				Coverage (%)	93.8	98.8	95.7
				95%CI	$(91.8, 95.7)$	$(97.8, 99.7)$	$(94.2, 97.3)$
0	0	1.0	0.760	Bias($\times 10^3$)	-61	-50	58
				95%CI	$(-65, -58)$	$(-54, -46)$	$(52, 64)$
				MSE($\times 10^4$)	67	50	134
				95%CI	$(62, 72)$	$(47, 53)$	$(124, 145)$
				Coverage (%)	77.7	89.7	98.3
				95%CI	$(10.3, 15.6)$	$(75.1, 80.3)$	$(97.5, 99.1)$

* MW: Mann-Whitney estimate (i.e., AUC_{obs}); R: Reiser's (2000) method; P: probit method.
** Simulation III was run 100 times. Each time, we generated 1000 simulated data sets. Each data set consists of 50 cases and 50 controls. each subject provides two replicate biomaker scores. Both true values and random errors were generated from log normal distributions with $\sigma_X^2 = 2$, $\sigma_Y^2 = 1$, $\sigma_{e_X}^2 = \sigma_{e_Y}^2 = 1$.

Table S4: Bias, Mean Square Error (MSE), and Coverage for $AUC_{true}\,(\mu)$ from Simulation I** with 50 subjects per group, $\sigma_X^2/\sigma_Y^2 = 2$, and $\theta^2 = 3$

λ	μ_Y	μ_X	AUC_{true}		MW*	R*	P*
0	0	0.25	0.570	Bias($\times 10^3$)	-29	3	7
				95%CI	$(-34, -24)$	$(-5, 10)$	$(-5, 19)$
				MSE($\times 10^4$)	42	96	193
				95%CI	$(37, 47)$	$(83, 110)$	$(164, 223)$
				Coverage(%)	91.8	97.3	98.0
				95%CI	$(89.4, 94.1)$	$(95.8, 98.8)$	$(96.7, 99.3)$
0	0	0.50	0.638	Bias($\times 10^3$)	-56	5	13
				95%CI	$(-61, -52)$	$(-3, 12)$	$(2, 25)$
				MSE($\times 10^4$)	65	94	184
				95%CI	$(58, 72)$	$(81, 107)$	$(155, 213)$
				Coverage (%)	82.7	97.1	97.8
				95%CI	$(79.6, 85.7)$	$(95.7, 98.6)$	$(96.5, 99.2)$
0	0	1.0	0.760	Bias($\times 10^3$)	-105	6	19
				95%CI	$(-108, -101)$	$(1, 12)$	$(12, 27)$
				MSE($\times 10^4$)	141	83	148
				95%CI	$(132, 149)$	$(75, 91)$	$(134, 161)$
				Coverage (%)	49.4	96.8	97.4
				95%CI	$(46.3, 52.4)$	$(95.7, 97.9)$	$(96.3, 98.4)$

* MW: Mann-Whitney estimate (i.e., AUC_{obs}); R: Reiser's (2000) method; P: probit method.

** Simulation I was run 100 times. Each time, we generated 1000 simulated data sets. Each data set consists of 50 cases and 50 controls. Each subject provides two replicate biomaker scores. Both true values and random errors are assumed to come from normal distributions with $\sigma_x^2 = 2$, $\sigma_y^2 = 1$, $\sigma_\epsilon^2 = \sigma_\eta^2 = 4.5$.

Table S5: Bias, Mean Square Error (MSE), and Coverage for $AUC_{true}\,(\mu)$ from Simulation II** with 50 subjects per group, $\sigma_X^2/\sigma_Y^2 = 2$, and $\theta^2 = 3$.

λ	μ_Y	μ_X	AUC_{true}		MW*	R*	P*
0	0	0.25	0.570	Bias($\times 10^3$)	-29	-15	-7
				95%CI	$(-33, -26)$	$(-20, -10)$	$(-15, 2)$
				MSE($\times 10^4$)	42	59	172
				95%CI	$(39, 46)$	$(54, 65)$	$(154, 189)$
				Coverage(%)	91.6	95.5	97.4
				95%CI	$(89.7, 93.4)$	$(94.3, 96.7)$	$(96.4, 98.4)$
0	0	0.50	0.638	Bias($\times 10^3$)	-52	18	39
				95%CI	$(-56, -49)$	$(13, 23)$	$(28, 51)$
				MSE($\times 10^4$)	60	68	253
				95%CI	$(55, 65)$	$(59, 77)$	$(225, 282)$
				Coverage (%)	84.3	99.0	98.8
				95%CI	$(81.9, 86.6)$	$(98.4, 99.6)$	$(97.9, 99.6)$
0	0	1.0	0.760	Bias($\times 10^3$)	-135	-47	-10
				95%CI	$(-139, -131)$	$(-52, -42)$	$(-23, 3)$
				MSE($\times 10^4$)	216	90.8	228
				95%CI	$(205, 226)$	$(82, 100)$	$(197, 259)$
				Coverage (%)	28.8	88.8	95.7
				95%CI	$(25.9, 31.7)$	$(86.9, 90.7)$	$(94.0, 97.4)$

* MW: Mann-Whitney estimate (i.e., AUC_{obs}); R: Reiser's (2000) method; P: probit method.
** Simulation II was run 100 times. Each time, we generated 1000 simulated data sets. Each data set consists of 50 cases and 50 controls. Each subject provides two replicate biomaker scores. True values were generated from log normal distributions and random errors were generated from normal distributions with $\sigma_X^2 = 2$, $\sigma_Y^2 = 1$, $\sigma_{e_X}^2 = \sigma_{e_Y}^2 = 4.5$.

Table S6: Bias, Mean Square Error (MSE), and Coverage for $AUC_{true}\,(\mu)$ from Simulation III** with 50 subjects per group, $\sigma_X^2/\sigma_Y^2 = 2$, and $\theta^2 = 3$.

λ	μ_Y	μ_X	AUC_{true}		MW*	R*	P*
0	0	0.25	0.570	Bias($\times 10^3$)	-27	-59	27
				95%CI	$(-31, -23)$	$(-113, -6)$	$(14, 39)$
				MSE($\times 10^4$)	41	949	298
				95%CI	$(37, 44)$	$(765, 1132)$	$(268, 328)$
				Coverage(%)	92.1	98.9	99.1
				95%CI	$(90.4, 93.8)$	$(96.9, 100.1)$	$(98.5, 99.7)$
0	0	0.50	0.638	Bias($\times 10^3$)	-65	-115	18
				95%CI	$(-69, -61)$	$(-169, -62)$	$(5, 30)$
				MSE($\times 10^4$)	76	1047	283
				95%CI	$(70, 81)$	$(832, 1262)$	$(254, 312)$
				Coverage (%)	78.3	95.4	98.4
				95%CI	$(75.7, 81.0)$	$(91.6, 99.1)$	$(97.4, 99.3)$
0	0	1.0	0.760	Bias($\times 10^3$)	-139	-220	-12.8
				95%CI	$(-143, -135)$	$(-273, -166)$	$(-27, 2)$
				MSE($\times 10^4$)	228	1396	253
				95%CI	$(216, 239)$	$(1100, 1694)$	$(226, 280)$
				Coverage (%)	26.4	86.4	95.4
				95%CI	$(23.8, 29.1)$	$(79.6, 93.2)$	$(93.5, 97.3)$

* MW: Mann-Whitney estimate (i.e., AUC_{obs}); R: Reiser's (2000) method; P: probit method.
** Simulation III was run 100 times. Each time, we generated 1000 simulated data sets. Each data set consists of 50 cases and 50 controls. each subject provides two replicate biomaker scores. Both true values and random errors were generated from log normal distributions with $\sigma_X^2 = 2$, $\sigma_Y^2 = 1$, $\sigma_{e_X}^2 = \sigma_{e_Y}^2 = 4.5$.
